# Temporal and spatial changes of cells positive for stem-like markers in different compartments and stages of human colorectal adenoma-carcinoma sequence

**DOI:** 10.18632/oncotarget.17330

**Published:** 2017-04-21

**Authors:** Guanglin Cui, Gang Xu, Li Zhu, Zhigang Pang, Wei Zheng, Zhenfeng Li, Aping Yuan

**Affiliations:** ^1^ Research Group of Gastrointestinal Diseases, the Second Affiliated Hospital of Zhengzhou University, Zhengzhou, Henan, China; ^2^ Faculty of Health, Nord University, Levanger, Norway

**Keywords:** stem-like marker, tumorigenesis, colorectum

## Abstract

Considerable evidence supports the idea that stem-like cells may play an essential role during the development of colorectal cancer (CRC). To accomplish this aim, we use immunohistochemistry (IHC) and double IHC with different potential stem-like markers, anti-musashi (Msi), anti-CD133, anti- LGR5 and anti-ALDH1 to examine the presentation of stem-like cells in different compartments including adenoma/CRC epithelium, transitional crypts and tumor stroma in colorectal adenoma and CRC. The results showed that cells positive for stem-like markers were remarkably increased in number and frequently observed in the adenoma/CRC epithelium, transitional crypts and tumor stroma. Notably, the population of cells positive for stem-liker markers was expanded from the base to the middle part of the transitional crypt in both adenoma and CRC tissues, reflecting that stem-like cells are likely involved in the process of colorectal tumorigenesis. Counting results showed that the grading scores of cells positive for LGR5 and ALDH1 in the adenoma/CRC epithelium were significantly increased relative with the control epithelium, and associated with the degree of dysplasia in the adenoma and node involvement in the CRC (all *P* < 0.05). In addition, the density of cells positive for stem–like markers in the adenomatous/cancerous stroma was also increased and paralleled an increase in the density of proliferative stromal cells labeled by PCNA, which were primarily identified as vimentin positive fibroblasts. Our results have revealed a changed temporal and spatial presentation of stem-like markers in different stages of human colorectal adenoma-carcinoma sequence, which might be a hallmark of the adenoma-carcinoma transition.

## INTRODUCTION

Colorectal cancer (CRC) is the fourth most frequent cancer and has a high mortality rate worldwide. Most CRCs, according to the adenoma-carcinoma sequence theory, develop from preformed adenoma polyps. The malignant potential of an adenomatous polyp is associated with its size, dysplastic degree and severity of atypia. This adenoma-carcinoma sequence provides an ideal opportunity to investigate the changes that occur during the development of CRC. Although many molecular, genetic and immunological alterations that are involved in this sequence have been identified [1–3], the exact mechanisms of the adenoma-carcinoma transition are still not fully understood.

Recent studies have provided considerable evidence to support the hypothesis that human tumors are derived from a subpopulation of cells with stem-like cell properties, termed tumor stem cells [1, 2]. Tumor stem-like cells have the capacity to self-renew, differentiate and drive tumorigenic growth [2]. The existence of tumor stem-like cells in precancerous adenomas as well as in CRCs have been described by several groups [2–10]. An association between the tumor stem-like cells and the prognosis of patients with CRC has also been suggested [11, 12]. During cancer initiation, the changes of tumor stem-like cells from precancerous to cancerous lesions are of particular interest because the alternation of tumor stem-like cells may be one of the hallmarks for colorectal tumorigenesis [10, 13, 14]. The histological identification of stem-like cells in either tumor or non-tumor tissues relies largely on specific markers. Several putative markers i.e., Musashi (Msi), CD44, CD133, leucine-rich repeat-containing G-protein-coupled receptor 5 (LGR5) and aldehyde dehydrogenase 1 (ALDH1) have been used to identify tumor stem-like cells in CRCs [2–9]. However, previous studies have primarily examined the presentation of stem-like markers in established adenomatous/cancerous epithelium; temporal and spatial variation of stem-like markers in different stages of colorectal adenoma-carcinoma sequence have not been well studied.

Given the above background, we hypothesized that an altered temporal and spatial presentation of stem-like markers could be an important hallmark that reflects the ongoing tumorigenesis in colorectum. In this study, we have therefore used different stem-like markers, Msi, CD133, LGR5 and ALDH1 to investigate the presentation of stem-like cells in the different compartment and histological stages of the colorectal adenoma–carcinoma sequence.

## RESULTS

### Stem-like markers in the adenomatous/cancerous epithelium

**Figure 1:**
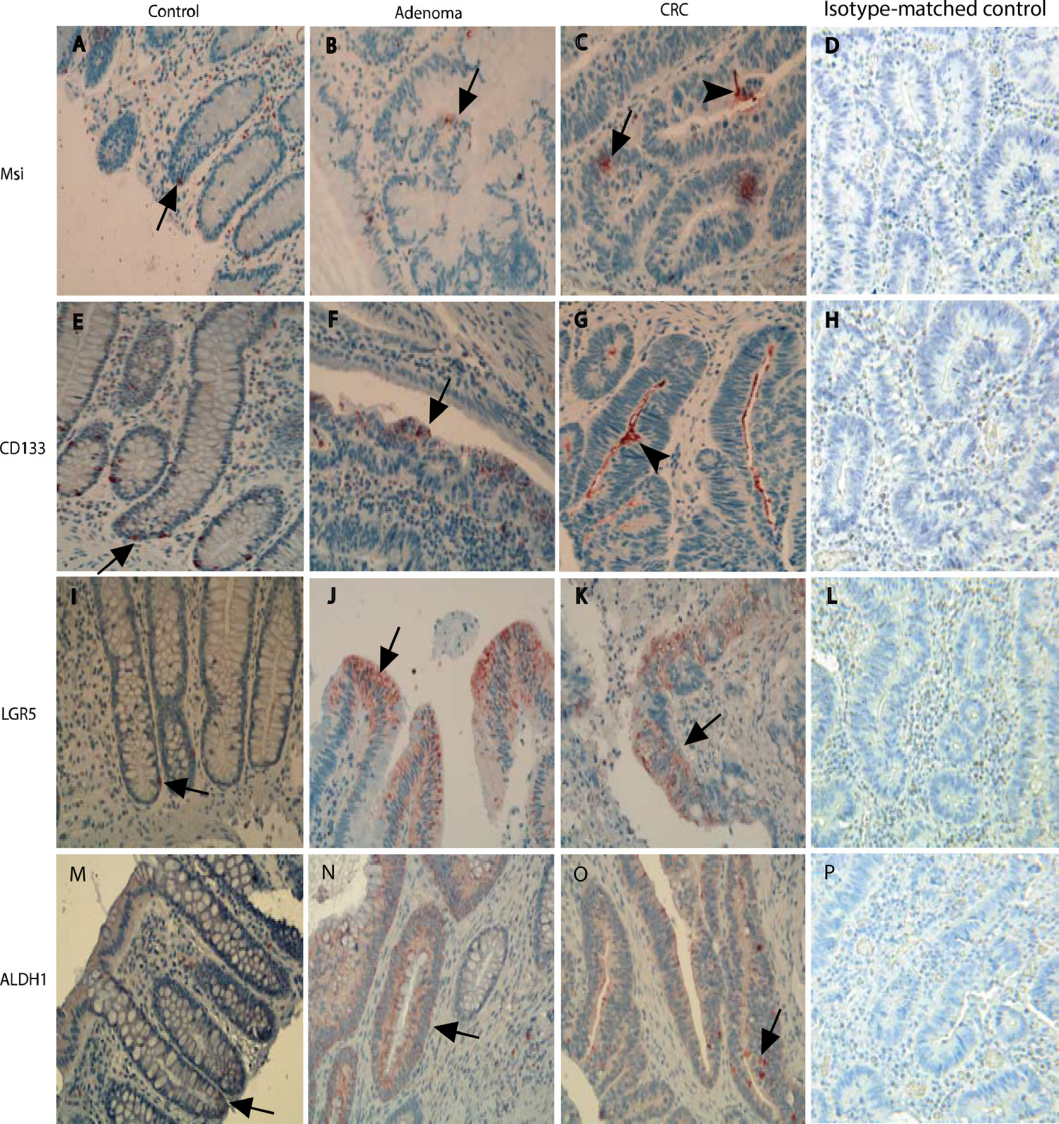
The presentation of stem-like markers Msi, CD133, LGR5 and ALDH1 in different stages of the adenoma-carcinomas evaluated with IHCs In normal controls, a few cells positive for stem-like markers were shown at the base of normal colonic crypts (Figure **1A**, **1E**, **1I** and **1M**). In the adenomatous epithelium, the cells positive for stem-like markers were expressed as a patchy destruction pattern in the surface of adenomatous crypt (Figure **1B**, **1F**, **1J** and **1N**), In the CRC epithelium, the cells positive for stem-like markers were also distributed in a diffusely pattern (Figure **1C**, **1G**, **1K** and **1O**). In sections with isotype-matched negative control antibodies staining (Figure **1D**, **1H**, **1L** and **1P**), no any positive cells were found.

The presence of stem-like markers in different stages of the adenoma-carcinoma sequence was evaluated with IHC using Msi, CD133, LGR5 and ALDH1 antibodies (Figure 1). The results showed that in normal controls, a few stem-like marker (Msi, CD133, LGR5 and ALDH1) positive cells were at the base of normal colonic crypts (Figure 1A). In the adenomatous epithelium, the cells positive for stem-like markers were expressed in a patchy distribution pattern on the surface of the adenomatous crypt (Figure 1B), In the cancerous epithelium, the cells positive for stem-like markers were also distributed in a diffused pattern (Figure 1C) or in the surface of CRC epithelium (Figure 1F) as reported in the literature [5].

Because LGR5 and ALDH1 are the two most promising and established stem-like markers in adenoma/CRC, we examined the immunoreactivity of LGR5 and ALDH1 in the adenoma/CRC epithelium with semi-quantitative analysis. The percentage of cells positive for LGR5 and ALDH1 in the adenomatous epithelium was 39.47% (15/38) and 47.37% (18/38), respectively. Additionally, the percentage of cells positive for LGR5 and ALDH1 in the CRC epithelium was 63.33% (19/30) and 66.67% (20/30), respectively. The positive rate for LGR5 and ALDH1 in the CRC epithelium were slightly higher than that in the adenomatous epithelium (both *P* > 0.05).

Then, the grading scores of LGR5 positive and ALDH1 positive cells in the adenomatous/cancerous epithelium were analyzed against clinical pathological parameters in adenomas and CRCs. In the adenoma, the grading scores of LGR5 positive and ALDH1 positive cells were correlated with degree of dysplasia (low-grade dysplasia vs. high-grade dysplasia; LGR5: 1.26 ± 0.10 vs. 166 ± 0.12; ALDH1: 1.52 ± 0.11 vs. 1.98 ± 0.14; both *P* < 0.05 by the Mann–Whitney test), but not with histological types (data not shown). In the CRC, the grading scores of both LGR5 positive and ALDH1 positive cells were associated with node involvement (node negative vs. node positive: LGR5, 1.44 ± 0.13 vs. 2.18 ± 0.24; ALDH1, 1.74 ± 0.13 vs. 2.40 ± 0.20: both *P* < 0.05 by the Mann–Whitney test). Both of LGR5 and ALDH1 grading scores were higher in CRC patients with advanced TNM stage than those with early stage (TNM stage I vs. II vs. III + VI: LGR5, 1.40 ± 0.17 vs. 1.43 ± 0.15 vs. 2.27 ± 0.26, *P* < 0.05; ALDH1, 1.60 ± 0.19 vs. 1.83 ± 0.16 vs. 2.29 ± 0.19, *P* > 0.05; both by the Kruskal-Wallis test).

### Changed presentation pattern of cells positive for stem–like markers in the transitional crypts close to adenomas and CRCs

**Figure 2:**
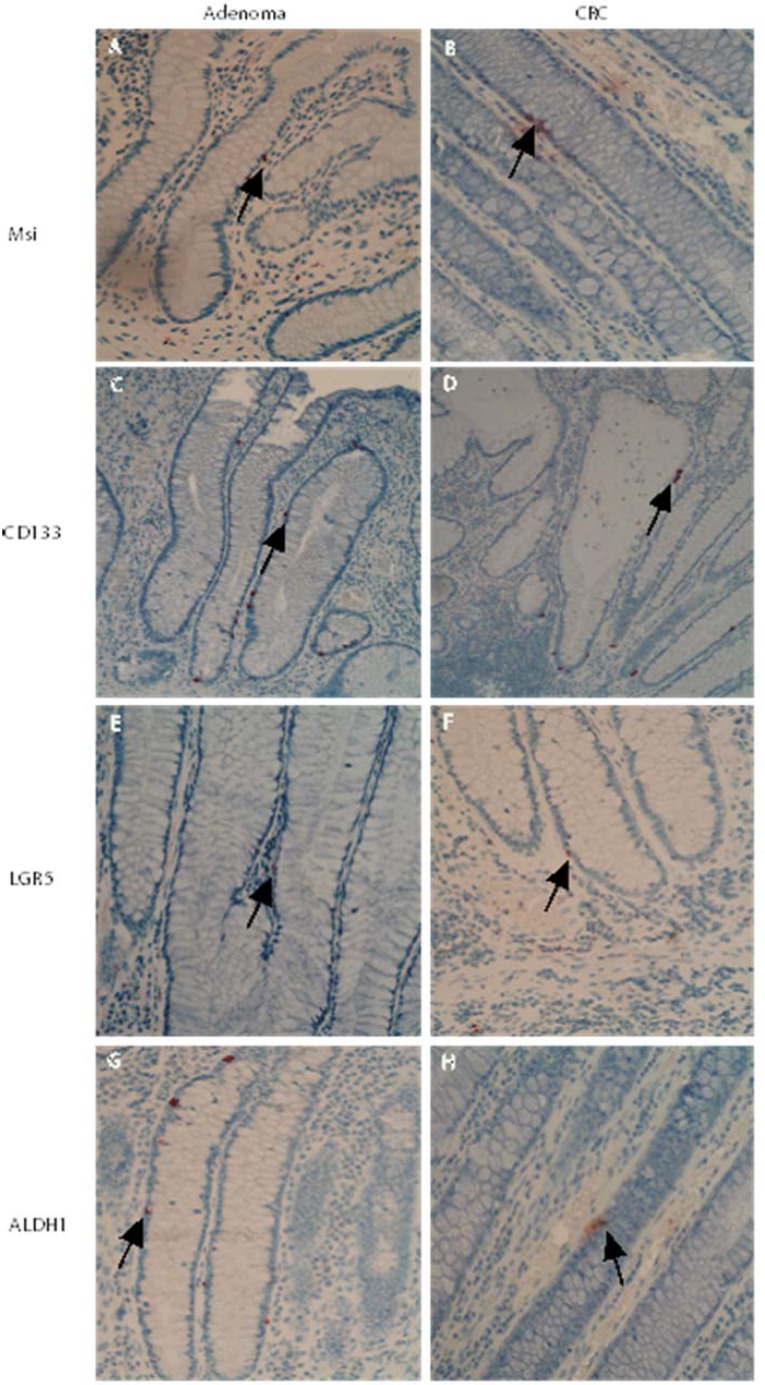
Changed presentation pattern of stem–like markers in the transitional crypts close to adenomas and CRCs In transitional crypts close to adenomas and CRCs, a changed presentation pattern of stem–like markers was observed. The populations of cells positive for stem-like markers Msi, CD133, LGR5 and ALDH1 were expanded, often from the base to the middle part, or even higher part of the transitional crypt (transitional crypt in adenomas, see Figure **2A**, **2C**, **2E** and **2G**; transitional crypt in CRCs, see Figure **2B**, **2D**, **2F** and **2H**).

Notably, in those sections with transitional crypts (6 of 30 adenoma sections, 5 of 30 CRC sections), a changed presentation pattern of cells positive for stem–like markers (Msi, CD133, LGR5 and ALDH1) was observed (see Figure 2). The location of cells positive for stem-like markers Msi, CD133, LGR5 and ALDH1 was expanded, often from the base to the middle part and occasionally to the top of the transitional crypt close to adenomas and CRCs (transitional crypt in adenomas, see Figure 2A, 2C, 2E and 2G; transitional crypt in CRCs, see Figure 2B, 2D, 2F and 2H). Those observations strongly suggested that expanding pathway of stem-like cells is from the base to the top during the development of adenomas and CRCs.

### Increased mesenchymal cells positive for stem-like markers in adenomatous/cancerous tumor stroma

**Figure 3:**
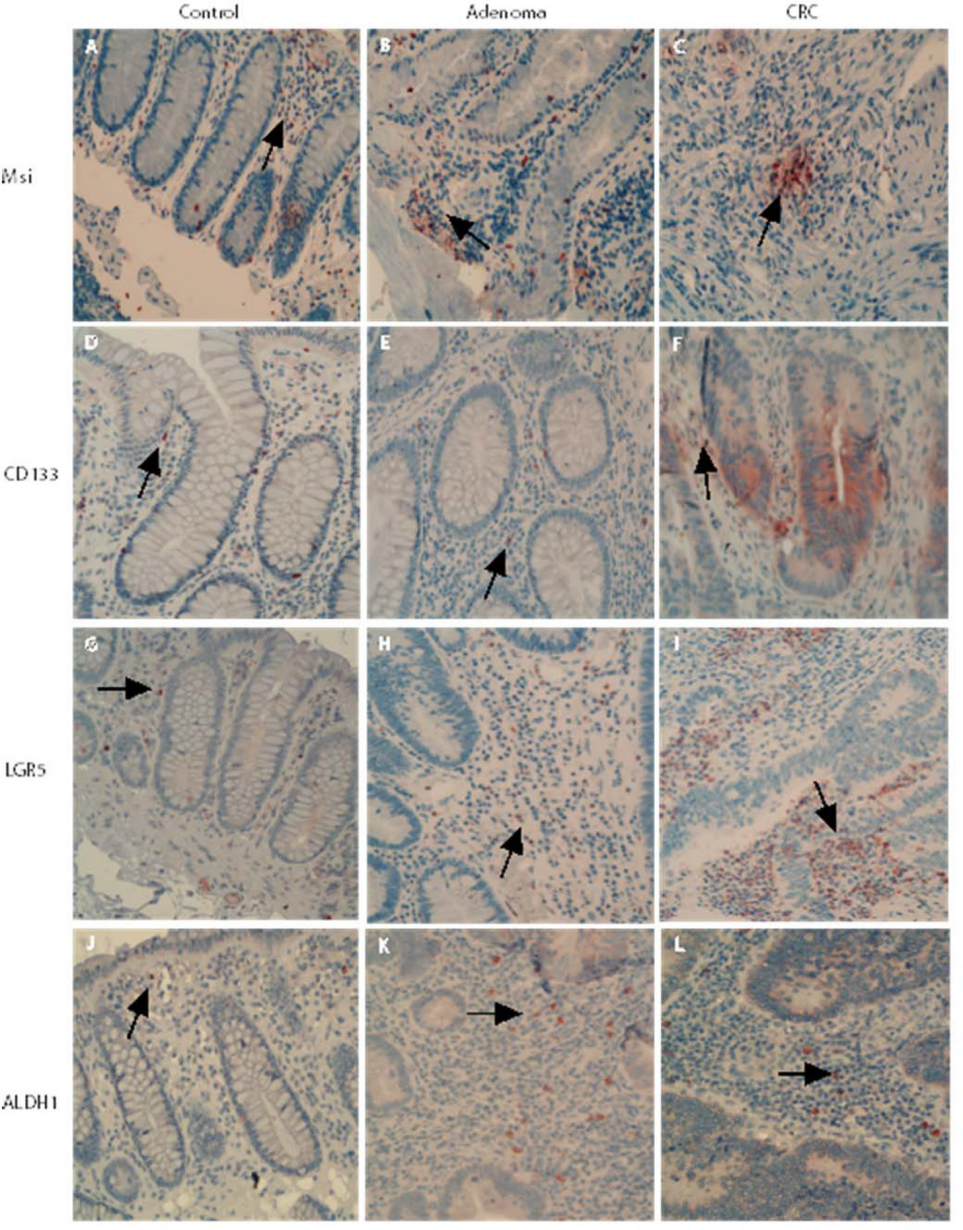
Mesenchymal stem-like markers in the adenomatous/cancerous tumor stroma The stromal cells positive for stem-like markers were diffusely distributed and the numbers of cells positive for stem-like markers Msi, CD133, LGR5 and ALDH1 in normal lamina propria and the densities were higher than that in the crypt (Figure **3A**, **3D**, **3G** and **3J**). In both the adenomas and CRC stroma, the cells positive for stem-like markers were increased and diffusely distributed in the tumor stroma between adenomatous (Figure **3B**, **3E**, **3H** and **3J**) or CRC (Figure **3C**, **3F**, **3I** and **3L**) epithelium.

**Figure 4:**
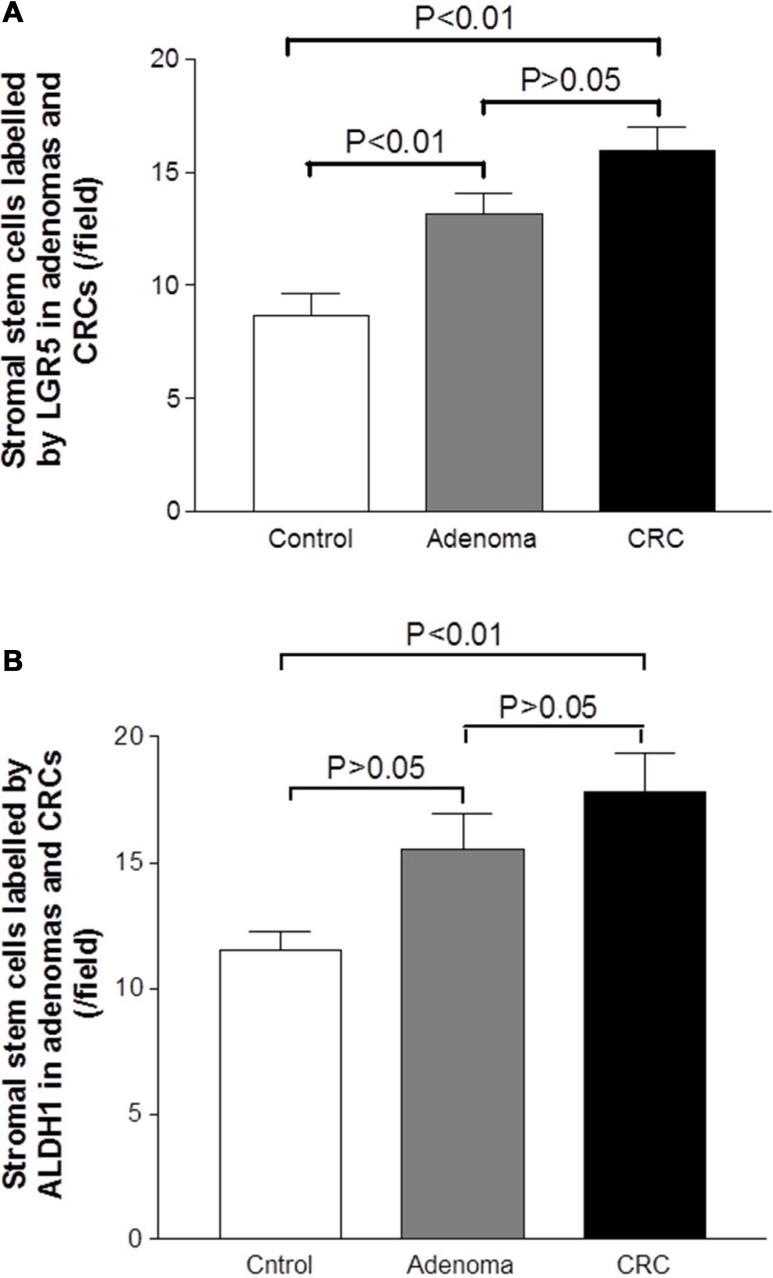
Graphic analysis of the density of mesenchymal stem-like cells labeled by LGR5 and ALDH1 in the adenomatous/cancerous tumor stroma The densities of mesenchymal stem-like cells labeled by LGR5 (Figure **4A**) and ALDH1 (Figure **4B**) were increased in the adenoma and CRC sections relative with the control section, that began from the adenoma lesion and even higher in the CRC lesion.

Mesenchymal stem-like cells resided in the tumor stroma play a critical role in forming a supportive environment for cancer growth and metastasis [21, 22]. In normal controls, the cells positive for stem-like markers could be observed in the lamina propria. The cells positive for stem-like markers Msi, CD133, LGR5 and ALDH1 were diffusely distributed, and the density of positive cells in lamina propria was higher than that in the crypt (Figure 3A, 3D, 3G and 3J). In adenoma tissues, the cells positive for stem-like markers were diffusely distributed in the tumor stroma between adenomatous epithelium (Figure 3B, 3E, 3H and 3J). In CRC tissue, the distribution pattern of cells positive for stem-like markers in tumor stroma (see Figure 3C, 3F, 3I and 3L) was very similar to that in adenomas. However, counting results showed that the densities of cells positive for stem-like markers LGR5 (Figure 4A) and ALDH1 (Figure 4B) in CRC tumor stroma were slightly higher than that in adenomas.

Then, the grading scores of LGR5 positive and ALDH1 positive cells in the adenomatous/cancerous stroma were analyzed against clinical pathological parameters. In the adenoma stroma, the grading scores of LGR5 positive and ALDH1 positive cells were not correlated with degree of dysplasia or histological types (data not shown). In the CRC stroma, the grading scores of LGR5 positive and ALDH1 positive cells were not associated with TNM stages (NM stage I vs. II vs. III + VI: LGR5, 16.86 ± 1.87 vs. 15.18 ± 1.52 vs. 6.11 ± 2.45, *P* > 0.05; ALDH1, 18.71 ± 2.69 vs. 20.36 ± 2.16 vs. 18.78 ± 2.15, *P* > 0.05; both by the Kruskal-Wallis test). However, we have found that although the difference did not reach statistical significance, the density grading scores were higher in CRC patients with node involvement than those without (node negative vs. node positive: LGR5, 14.89 ± 1.20 vs. 18.00 ± 2.19; ALDH1, 18.00 ± 1.37 vs. 22.22 ± 2.59; both *P* < 0.05 by the Mann–Whitney test).

### Increase in cells positive for stem-like markers in the adenomatous/cancerous stroma was accompanied by an increase in PCNA-positive proliferative cells that consisted primarily of fibroblasts

**Figure 5:**
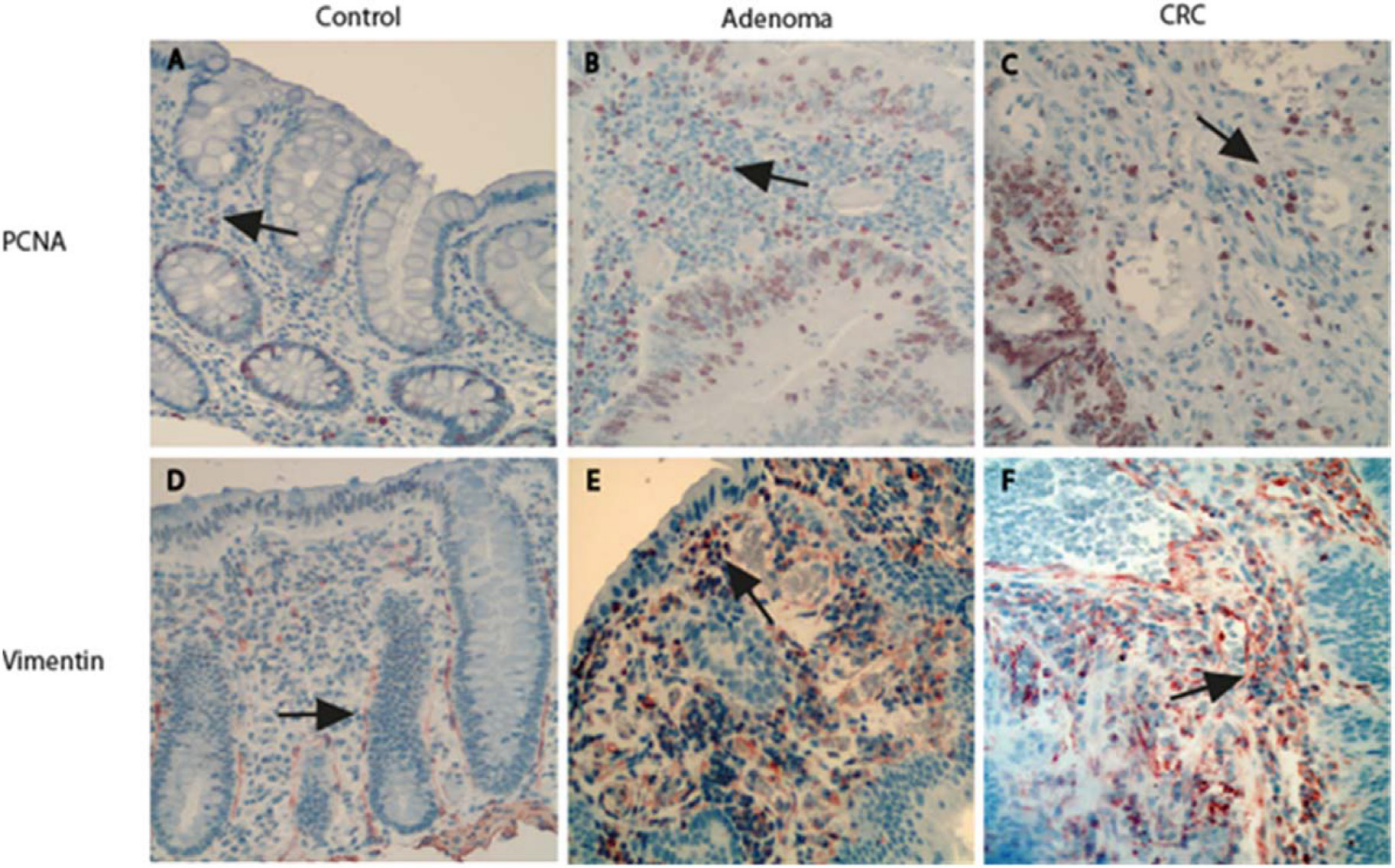

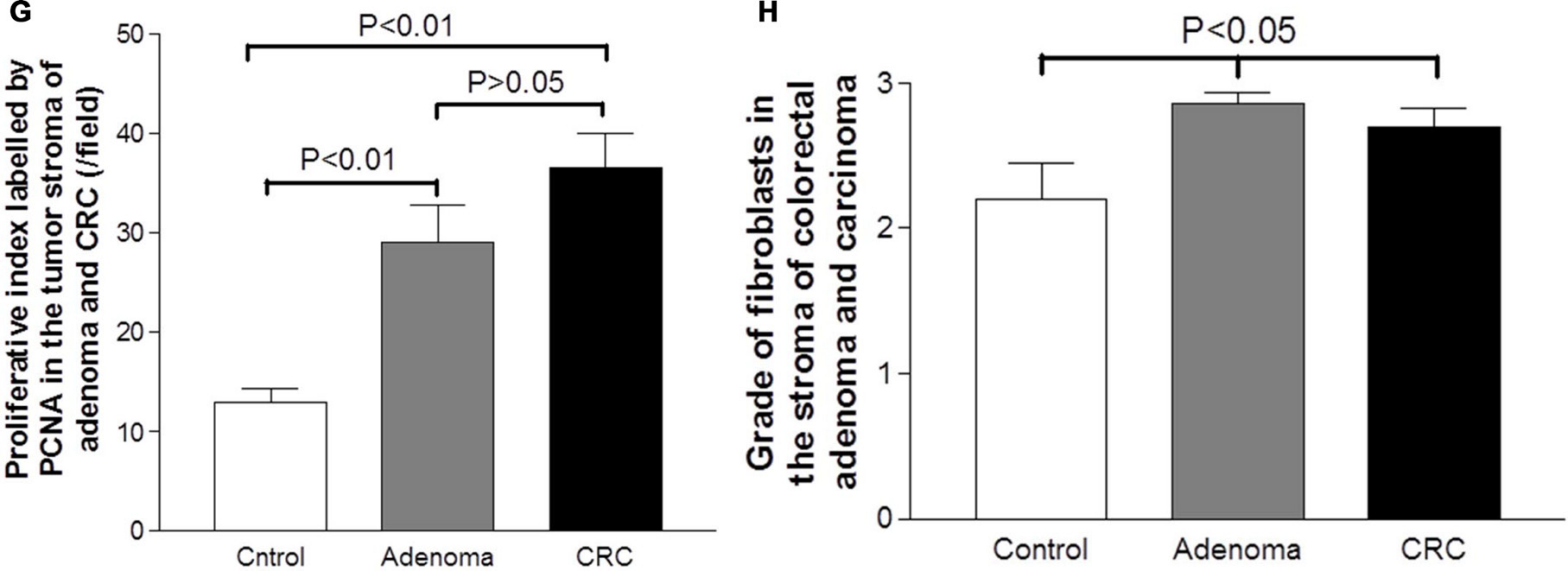
Increased proliferation rate of stromal cells that primarily identified as vimentin positive fibroblasts in adenomas and CRCs The proliferation rate in the tumor stromal cells was evaluated with PCNA IHC (Figure **5A**–**5C**). The stromal cells positive for PCNA in both the adenoma and the CRC were significantly increased relative to the control (Figure **5G**) (both *P* < 0.0001). Further IHC examination revealed that fibroblasts labeled by vimentin were observed within both the pericryptal and non-pericryptal regions in the stroma of control (Figure **5D**), adenoma (Figure **5E**) and CRC (Figure **5F**). When the density of fibroblasts was graded, the grading scores were shown in a non-statistically increased in the adenoma (Figure **5H**, *grey bar*) and CRC stroma (Figure 5H, *black bar*) relative to the control (Figure 5H, *white bar*).

Tumor stroma and contributes to the cancer initiation and progression [23]. We therefore investigated proliferation activity of stromal cells in the adenomatous/CRC stroma. Along with the increase in cells positive for stem-like markers in the tumor stroma, the number of proliferative cells in the adenomatous/cancerous tumor stroma was also significantly increased. The proliferation rate in the tumor stromal cells was evaluated with PCNA IHC (Figure 5A–5C). The results showed that the proliferation rate increased in both adenomatous and cancerous tumor stromal cells compared to controls (Figure 5G) (both *P* < 0.0001 by the Mann–Whitney test). No significant increase in proliferation rate was observed between adenoma and CRC stroma (*P* > 0.05).

Because fibroblasts are the main type of stromal cells and the activation of stromal fibroblasts are involved in the initiation and progression of CRC [23, 24], we examined the expression of fibroblasts labeled with vimentin with IHC and the proliferative activity of vimentin positive fibroblasts with double IHC in the adenoma/CRC stroma. The results of vimentin IHC showed that fibroblasts were observed in the whole stroma within both the pericryptal and non-pericryptal regions in adenoma, CRC and control group sections (Figure 5D–5F). In the adenoma section, dense fibroblasts were observed in the whole stroma but were particularly dense in the peri-adenomatous epithelium region (Figure 5E) compared to controls (Figure 5D). In the CRC sections, the presentation of fibroblasts was similar to that of the adenomas and many fibroblasts could be found in the tumor stroma (Figure 5F). When the density of vimentin positive fibroblasts was graded, the grading scores were higher in adenomas (Figure 5H, grey bar) and CRCs (Figure 5H, black bar) relative to controls (Figure 5H, white bar), although statistical significance was not reached.

**Figure 6:**
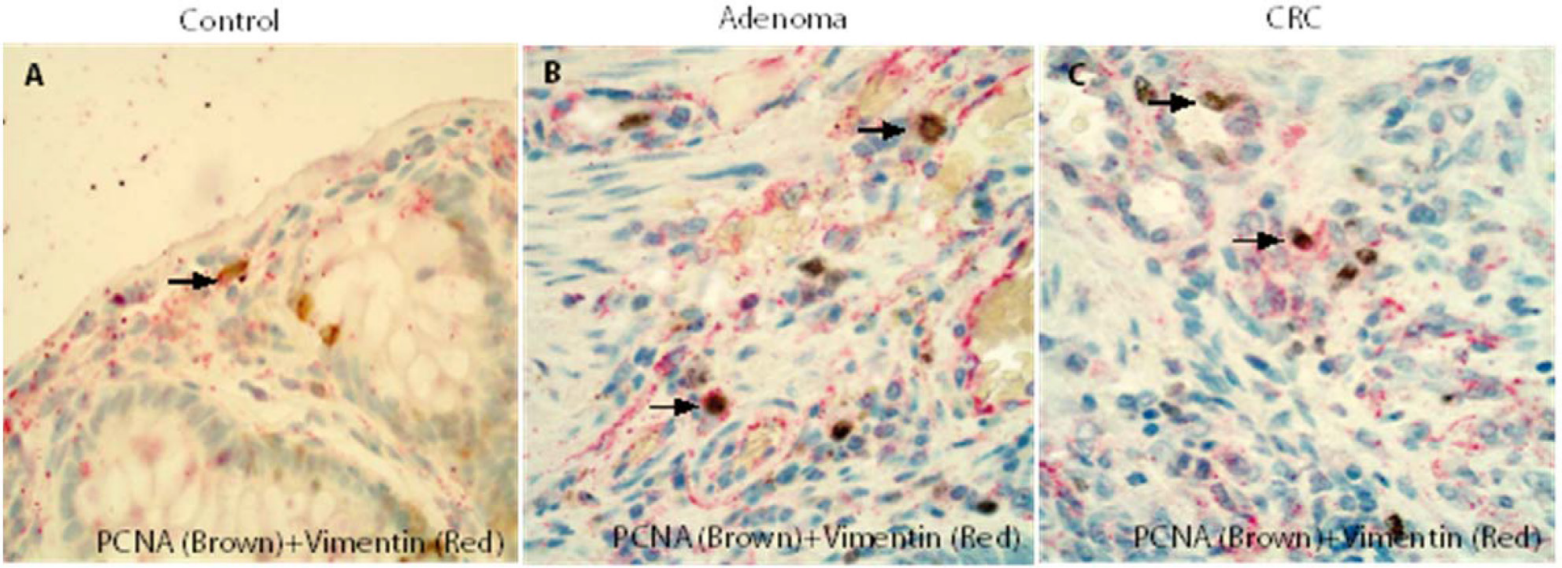
Double IHCs confirmed that fibroblasts in the adenoma/CRC stroma might have a high proliferative capacity The results of double IHC demonstrated that many of PCNA positive stromal cells in the control (Figure **6A**), adenomas (Figure **6B**) and CRCs (Figure **6C**) were fibroblasts.

The results of double IHC demonstrated that many of the PCNA positive cells in the control (Figure 6A), adenoma (Figure 6B) and CRC (Figure 6C) stromal cells were vimentin positive fibroblasts and confirmed that vimentin positive fibroblasts in the adenoma/CRC stroma might have a high proliferative capacity.

## DISCUSSION

In this study, we examined the presentation pattern of stem-like markers in different stages and compartments of the adenoma-carcinoma sequence and translational crypts adjacent to adenomas and CRCs. We were able to demonstrate an altered localization and expression pattern of cells positive for stem-like markers in those tissues. In addition, we have also observed an increase in cells positive for stem-like markers in the adenoma/CRC stroma that accompanied by an active proliferation rate in the tumor stromal cells, and many of the proliferative stromal cells were identified as vimentin positive fibroblasts. These findings suggest that the change in the presentation pattern of the stem-like markers in different stages and compartments of the adenoma-carcinoma sequence reflects an ongoing tumorigenesis. Such change might be one of the hallmarks of colorectal tumorigenesis and related to the stromal response that resulted from tumorigenesis.

Under physiological conditions, only a low density of cells positive for stem-like markers reside in the base of crypts, and they play an important role in maintaining normal tissue homeostasis and function. Thus, it is not unusual that the number of cells positive for stem-like markers in the normal crypt and lamina propria was low in control tissues. However, when colorectal mucosa becomes tumorigenic, the stem-like cells are activated and the number of cells positive for stem-like markers was significantly increased. In the current study, we observed a large change in the presentation of stem-like markers in different compartments of adenoma and CRC tissues. The cells positive for various types of stem-like markers were observed in both the adenomatous/cancerous epithelium and the tumor stroma. The cells were mostly distributed in a patchy pattern in the adenomatous/cancerous epithelium. This finding might reflect the fact that stem cells are substantially activated during the procession of colorectal tumorigenesis.

The importance of stem cells in the initiating of adenomas has been recognized [2, 5, 11]. Several potential stem-like markers have been used to identify stem cells in adenomas/CRC. LGR5 and ALDH1 are the two most promising and established stem-like markers and have been frequently used [3, 5, 7, 10, 15]. Studies have reported that LGR5 and ALDH1 expression can reflect the changes in stem-like cells during the process of colorectal tumorigenesis. However, whether the mechanism is top-down or bottom-up is a matter of debate [11, 16]. Some studies suggest that dysplastic cells are routinely found at the luminal surface of adenomatous crypts that contain genetic alterations in the adenomatous polyposis coli (APC) gene and neoplasia-associated patterns of gene expression. Stem-like cells are also observed at the luminal surface of adenomatous crypts. Thus, these findings imply that the development of adenomatous polyps proceeds through a top-down mechanism. Alternatively, genetically-altered cells in the superficial portions of the mucosae spread laterally and downward to form new crypts that first connect to preexisting normal crypts and eventually replace them [17]. However, evidence from other studies has supported the idea that colorectal polyps are polyclonal and arise from a combination of host genetic features via a bottom-up mechanism [18, 19]. The transitional mucosa is characterized morphologically by an increase in mucosal thickness, lengthening of the crypts and goblet cell hyperplasia. Since transitional mucosa reflects the ongoing histopathological procession of tumorigenesis, studying the changes of pathological aspects inside crypts might provide critical information for the understanding of tumorigenesis procession. Indeed, several groups including ours have demonstrated the histological, genetic, histochemical and immunological changes in the mucosa adjacent to the adenoma and CRC epithelium [20–25]. However, there is very limited information available regarding the change of stem-like cells in the transitional mucosa during the development of CRC. To our best knowledge, the current study is the first of its kind to demonstrate the temporal and spatial changes of stem-like markers in the transitional mucosa from colorectal adenomas to sporadic CRCs. We have observed an altered presentation pattern of cells positive for stem-like markers in the transitional mucosa close to adenomas and CRCs. In controls, cells positive to stem-like markers were only located at the base of the crypts. However, some of positive cells were located in the middle part and occasionally expanded to the top of the transitional crypt close to adenomas and CRCs. In light of our results, such altered location of cells positive for stem-like markers in the transitional mucosa may reflect the undergoing expanding path of stem-like cells inside preneoplasic crypts and could be the supportive evidence for the bottom-up hypothesis. This information could also be very useful as an indicator of a malignancy potential and as a target for novel therapeutic approaches.

With respect to the relationship between stem-like cells (labeled with LGR 5 and ALDH1) and clinicopathological parameters of patients with adenomas and CRCs, we have observed slight increased scores of LGR5 and ALDH1 positive cells in the adenomatous epithelium with high degree of dysplasia. We have also found that grading scores of LGR5 and ALDH1 positive cells in the cancerous epithelium were significantly increased in CRC patients with advanced TNM stages than those with early stages. Those observations are consist with other groups’ findings [15, 26–32], and imply that increased stem-like cell density might be the potential predicators for disease stage progression in both the adenoma and CRC. Mesenchymal stem like cell can be observed in the tumor stroma, it plays a critical role in remodeling tumor stroma and forming a supportive environment for cancer growth and metastasis [33]. In this study, many mesenchymal cells positive for these potential stem-like markers could be observed in the adenoma/CRC stroma, which was accompanied by an increase in proliferation in the stromal cells that has been previously reported by our group [34]. When the cell positive for stem-like markers was analyzed against the clinical pathological parameters in the adenomatous/cancerous stroma, we have observed that the grading score of cell positive for LGR5 and ALDH1 was not associated with most clinical pathological parameters i.e. degree of dysplasia and histological types in the adenoma and TNM stage in the CRC. However, the density grading scores of positive cell were higher in CRC patients with node involvement than those without, although the difference did not reach statistical significance. This observation suggested that the tumorigenesis may results in a general stem-like cell response in the tumor stroma, such activation might contribute to the diseases progression.

Since vimentin-expression is one of the hallmarks of epithelial-mesenchymal-transition (EMT) during the progression of CRC, we have therefore further examined the activity of vimentin positive fibroblasts in the adenomatous/cancerous stroma. The results showed that these PCNA positive proliferative stromal cells were mostly identified as vimentin positive fibroblasts in the adenoma/CRC stroma. As illustrated by many studies [1, 34–36], fibroblasts are the main type of stromal cells in adenoma and CRC tissue and they promote the progression of adenoma to CRC. The exact mechanisms for the elevated proliferation seen in tumor stromal fibroblasts are thus far undetermined. It has been reported that vimentin-expression is one of the hallmarks of EMT during the progression of CRC and vimentin positive stromal cells may be with tumorigenic cell characteristics and play a vital role during the invasion of CRC. In addition, many factors are related to the stromal activation during the development of human cancers [14, 35]. For instance, the release of cytokines from the tumor microenvironment may participate in the modulation of the proliferative activity of the stromal cell [37]. In our previous studies, we have demonstrated an elevated expression pattern of IL-17A in the tumor microenvironment of adenoma/CRC [25, 38, 39]. It has been found that IL-17A stimulates stem-like cells and promote the development of various types of cancers [40–43]. Our current findings may provide further evidence to support the notion that the activation of vimentin positive fibroblasts in the adenomatous/cancerous stroma might be one of the hallmarks of EMT and are involved in the process of colorectal tumorigenesis.

In summary, this study showed a temporal and spatial variation of stem-like markers in different compartments and stages of the colorectal adenoma-carcinoma sequence, including adenoma/CRC epithelium, transitional crypts and tumor stroma, throughout all stages of the colorectal adenoma-carcinoma sequence. Such dynamic change of stem-like markers reflect the ongoing tumorigenesis and may be a hallmark of the adenoma-carcinoma transition.

## MATERIALS AND METHODS

A total of 30 biopsies of colorectal adenomas excised completely by endoscopic polypectomy (male/female ratio 22/8; age 36–70 years) and 30 biopsies of CRC excised by surgery (male/female ratio 17/13; age 49–70 years) were included in this study. No patients received radiotherapy and/or chemotherapy preoperatively. Moreover, biopsies from 12 subjects (male 8, female 4, ages 35–60 years) with normal colonoscopy and histology served as a normal control group. All biopsies were prepared and embedded in paraffin routinely. Sections were cut at a thickness of 4 μm and then stained with hematoxylin and eosin (*H&E*). Basic information was summarized in Table 1. This study was approved by the local Medical Research Committee.

**Table 1:** Basic histological information of patients and normal individuals

	*N*	Location		Histopathology		Dysplasia degree		
		colon	rectum	tubular	tubulovillous	LGD		HGD
Normal	12	5	7					
Adenoma	30	20	10	20	10	18		12
						TNM stage		
				adenocarcinoma		I	II	III
CRC	30	16	14	30		7	10	13

LGD: low grading of dysplasia.

HGD: high grading of dysplasia.

### Immunohistochemistry (IHC) for stem-like markers, stromal proliferative activity labeled by PCNA and stromal fibroblasts labeled by vimentin

Sections for IHC were deparaffinized in xylene, rehydrated in graded ethanol, and incubated in a 0.3% H_2_O_2_ solution in methanol for 15 minutes to block endogenous peroxidases. Antigen retrieval was achieved by boiling sections for 15 minutes in 0.01 M citrate buffer, pH 6.0. Nonspecific binding was blocked by incubating sections in phosphate buffered saline (PBS) containing 4% normal bovine serum and 0.25% Triton-X 100. The slides were rinsed three times with PBS with 0.25% Triton-X 100 (PBS-T) for 5 min and incubated overnight at 4°C with different antibodies for putative stem-like markers: anti-Msi (DAKO, Carpinteria, CA, USA), anti-CD133 (Abcam, Cambridge, UK), anti-LGR5 (MBL International, Woburn, MA 01801, USA) and anti-ALDH1 (BD Bioscience, San Jose, CA, USA) individually. For PCNA and vimentin IHC, sections were incubated overnight at 4°C with primary antibodies: anti-PCNA polyclonal antibody (Abcam, Cambridge, UK) and anti-Vimentin monoclonal antibody (DAKO, Carpinteria, CA, USA), respectively. After primary antibody staining, the slides were washed with PBS-T for 10 minutes, and detection was performed using the Vectastain *Elite ABC* Kit (Vector Lab., Burlingame, CA, USA) according to the manufacturer's instructions and our published method [14, 15]. Next, 3-Amino-9-ethylcarbazole (AEC; Vector Laboratories, Burlingame, CA, USA) was used as the chromogen, and slides were counterstained with Mayer's hematoxylin. Negative control slides for IHC were made routinely: (1) primary antibodies were substituted with the isotype-matched control antibodies; and (2) a secondary antibody was substituted with PBS-T.

### Double IHC for the examination of proliferative activity in stromal fibroblasts

Because fibroblasts are the main type of tumor stromal cells [44], we examined the proliferative activity of fibroblasts with double IHC in the tumor stroma. Double IHC with PCNA/Vimentin (to label fibroblasts) antibodies was performed using the *EnVision* Doublestain System kit (DAKO, Carpinteria, CA, USA) according to the manufacturer's instructions and our published methods [45, 46]. In brief, the slides were incubated overnight at 4°C with anti-PCNA antibody after antigen retrieval and then incubated with labeled polymer-horseradish peroxidase-anti-mouse and anti-rabbit antibodies for 30 minutes at room temperature. Peroxidase activity was detected with the enzyme substrate 3,3′-diaminobenzidine tetrachloride (*DAB*). After quenching the enzyme reaction, the slides were incubated in Doublestain Block at room temperature for 5 minutes to block endogenous phosphatases. The slides were then incubated with anti-Vimentin antibody for 2 hours at room temperature. After washing, the slides were incubated with labeled polymer-alkaline phosphatase anti-mouse and anti-rabbit antibodies for 30 minutes at room temperature. A *Fast Red* chromogen substrate solution was used to visualize anti-vimentin antibody. The sections were slightly counterstained with Mayer's hematoxylin.

### Morphological evaluation

The semi-quantified density grading of stem-like positive cells was employed according to the method described in our previous publication [45, 47]. In brief, semi-quantitative scoring was conducted in at least five well-orientated fields with abundant positive cell distribution from each slide under 400 × high-power magnification. In the adenoma/CRC epithelium, the number of LGR5 positive and ALDH1 positive cells was graded on a scale of 0–3: grade 0 indicated an absence of LGR5/ALDH1 positive cells; grade 1 indicated a 1%–5% presence of LGR5/ALDH1 positive cells; grade 2 indicated a 6%–25% presence of LGR5/ALDH1 positive cells; and grade 3 indicated a 26%–100% presence of LGR5/ALDH1 positive cells. The results were grouped as positive (grade 2 or 3), or negative (grade 0 or 1), given that the normal gastric mucosa was identified as grade 1 for LGR5/ALDH1 expression [48]. In tumor stroma, fibroblasts (labeled by Vimentin) were found at a very high density and were evaluated with light microscopy using the following criteria described by Adegboyega et al. [49]: negative immunoreactivity (IR) (−), 1% to 25% positive cells (+), 25% to 50% positive cells (++), and greater than 50% positive cells (+++). The number of PCNA positive (nucleus) cells in the tumor stroma were counted in at least 5 optional fields with abundant distribution from each slide under a 400 × high-power magnification. The average values were used for statistical analysis.

### Statistical analysis

The results were expressed as the mean ± SEM unless otherwise stated. Statistical significance was evaluated by the Mann–Whitney and Kruskal–Wallis tests. Values of *P <* 0.05 were considered significant.
